# Increased Impulsivity Retards the Transition to Dorsolateral Striatal Dopamine Control of Cocaine Seeking

**DOI:** 10.1016/j.biopsych.2013.09.011

**Published:** 2014-07-01

**Authors:** Jennifer E. Murray, Ruth Dilleen, Yann Pelloux, Daina Economidou, Jeffrey W. Dalley, David Belin, Barry J. Everitt

**Affiliations:** a Department of Psychology, Addenbrooke’s Hospital, University of Cambridge, Cambridge, United Kingdom; b Behavioural and Clinical Neuroscience Institute, Cambridge, United Kingdom; c Department of Psychiatry, Addenbrooke’s Hospital, University of Cambridge, Cambridge, United Kingdom; d INSERM U1084-LNEC team Psychobiology of Compulsive Disorders, Universtié de Poitiers, Poitiers; e INSERM European Associated Laboratory, Poitiers; f Institute of Neuroscience de la Timone, University of Aix-Marseille, Marseille, France

**Keywords:** Cocaine, dopamine, drug addiction, goal-directed, habitual, striatum

## Abstract

**Background:**

Development of maladaptive drug-seeking habits occurs in conjunction with a ventral-to-dorsal striatal shift in dopaminergic control over behavior. Although these habits readily develop as drug use continues, high impulsivity predicts loss of control over drug seeking and taking. However, whether impulsivity facilitates the transition to dorsolateral striatum (DLS) dopamine-dependent cocaine-seeking habits or whether impulsivity and cocaine-induced intrastriatal shifts are additive processes is unknown.

**Methods:**

High- and low-impulsive rats identified in the five-choice serial reaction-time task were trained to self-administer cocaine (.25 mg/infusion) with infusions occurring in the presence of a cue-light conditioned stimulus. Dopamine transmission was blocked in the DLS after three stages of training: early, transition, and late-stage, by bilateral intracranial infusions of α-flupenthixol (0, 5, 10, or 15 μg/side) during 15-min cocaine-seeking test sessions in which each response was reinforced by a cocaine-associated conditioned stimulus presentation.

**Results:**

In early-stage tests, neither group was affected by DLS dopamine receptor blockade. In transition-stage tests, low-impulsive rats showed a significant dose-dependent reduction in cocaine seeking, whereas high-impulsive rats were still unaffected by α-flupenthixol infusions. In the final, late-stage seeking test, both groups showed dose-dependent sensitivity to dopamine receptor blockade.

**Conclusions:**

The results demonstrate that high impulsivity is associated with a delayed transition to DLS-dopamine-dependent control over cocaine seeking. This suggests that, if impulsivity confers an increased propensity to addiction, it is not simply through a more rapid development of habits but instead through interacting corticostriatal and striato-striatal processes that result ultimately in maladaptive drug-seeking habits.

Increasing evidence suggests that addiction results from the convergence of various neurobiological adaptations in vulnerable subjects, eventually resulting in the loss of control over maladaptive drug seeking (1–3). Exposure to addictive drugs, such as cocaine, not only impairs executive processes, resulting in impulse control deficits and behavioral inflexibility (4), but it also facilitates the development of drug-seeking habits (3,5,6), thereby rendering instrumental actions that are resistant to their immediate consequences and motivational significance (6,7). Addictive drugs trigger adaptations within corticostriatal circuitry, including reductions in metabolic activity and D2 dopamine receptors, that are initially restricted to the ventral limbic areas of the striatum and prefrontal cortex but eventually encompass the more dorsolateral, associative and cognitive, territories of these structures (8–10). This progressive shift from limbic to cognitive corticostriatal networks that occurs over the course of addiction (11) takes place alongside a transition from the nucleus accumbens to the dorsolateral striatum (DLS) in the locus of control over drug seeking and taking (12) and the associated imbalance in fronto-striatal and striato-striatal functional coupling (13) displayed by former and current addicted individuals.

Studies in animals have further demonstrated that this ventral to DLS shift in the control over drug seeking (14,15) is not only associated with the development of habitual responding for the drug as assessed by devaluation procedures (3,6) but also reflects the emergence of compulsive cocaine seeking (16). The latter, a hallmark feature of addiction (17), is predicted by the behavioral trait of high impulsivity (18), which is associated with low D2/3 dopamine receptor availability in the ventral striatum (19). This has led to hypotheses suggesting that impulsivity and habits, with their striatal dopaminergic substrates, interact during the development of cocaine addiction, but the neurobiological basis of this interaction is unknown. Neurocomputational learning theory-based, actor-critic models of basal ganglia function (20) suggest that high impulsivity and its associated low D2 dopamine receptor availability in the ventral striatum facilitate the transition to DLS control over drug self-administration. However, we and others have suggested that compulsive drug seeking in addiction might instead result from weak inhibitory control over a rather independently established, drug-influenced, maladaptive incentive habit (4,21).

We therefore directly investigated whether high impulsivity interacts with the recruitment of dopamine-dependent DLS control over cocaine-seeking behavior over an extended period of cocaine self-administration. To do this, we investigated the effects of bilateral infusions of the dopamine receptor antagonist α-flupenthixol into the DLS of rats identified as high (HI) and low impulsive (LI) in the 5-choice serial reaction-time task (5-CSRTT), on cue-controlled cocaine-seeking behavior at early, transitional, and late stages of training under a second-order schedule of reinforcement for cocaine (22). Under these conditions we have previously shown that cocaine seeking becomes dependent upon dopamine transmission in the DLS (14,18,23), and the functional recruitment of this dopaminergic mechanism is a neurobiological marker of the emergence of drug-seeking habits (3,6).

## Methods and Materials

### Subjects

Forty male Lister Hooded rats (Charles River Laboratories, Kent, United Kingdom) weighing approximately 300 g on arrival were housed as described previously (23). Experiments were conducted in accordance with the United Kingdom 1986 Animals (Scientific Procedures) Act.

### 5-CSRTT

**Apparatus and Procedure.** The 5-CSRTT apparatus has been described in detail elsewhere (24,25) (Supplement 1). The training procedure was identical to that previously described (18). Each training session began with illumination of the operant chamber by a house light and the delivery of a food pellet in the magazine. Pushing open the magazine panel and collecting this pellet initiated the first trial. After a fixed intertrial interval (ITI), a light at the rear of one of the response apertures was briefly illuminated. Responses in this aperture within a limited-hold period (5 sec) were reinforced by the delivery of a food pellet in the magazine (correct responses). Responses in a nonilluminated aperture were recorded as incorrect responses and were punished by a 5-sec time-out period. Failure to respond within the limited-hold period counted as an omission and was likewise punished. Additional responses in any aperture before food collection (perseverative responses) were recorded but not punished. Responses made in any aperture before the onset of the target stimulus, or premature responses, were punished by a 5-sec time-out period. Across training sessions, the ITI was gradually increased, and the stimulus duration was gradually decreased (25). Subjects were considered to have acquired the task when accuracy was > 75% and omissions were fewer than 20% while the stimulus duration was .5 sec with a 5-sec ITI.

After 2 weeks of stable responding, rats underwent three 60-min challenge 7-sec ITI (long intertrial interval [LITI]) sessions, separated by baseline 5-sec ITI sessions (18,26). The LITIs markedly increase premature responding, thereby facilitating the identification of interindividual differences in impulsivity. The number of premature responses during LITI sessions provides an index of impulse control (18,19,24–26), which is used to identify HI or LI rats. Subjects were ranked according to the mean number of premature responses during the last two LITI sessions (10,18). Those with <20 or >50 premature responses were selected as LI and HI rats, respectively (*n* = 8/group) (Figure S1 in Supplement 1).

In addition, premature responses, magazine panel pushes, correct and incorrect responses, omitted trials, and collection latency (milliseconds to collect the food pellet) were averaged across the baseline sessions preceding each of the last two LITI sessions to compare baseline behavioral performance in LI and HI rats.

### Surgery

Rats then underwent standard intravenous and intrastriatal surgeries under general anesthesia (Supplement 1). Cannulae were implanted bilaterally 2 mm above the dorsolateral striatum (anterior/posterior+1.2, medial/lateral±3, dorsal/ventral-3 [15]; AP and ML coordinates measured from bregma, DV coordinates from the skull surface, incisor bar at −3.3 mm [27]).

### Drugs

Cocaine hydrochloride (Macfarlan-Smith, Edinburgh, United Kingdom) was dissolved in sterile .9% saline. α-Flupenthixol (Sigma Aldrich, Poole, United Kingdom) was dissolved in double-distilled water. Drug doses are reported in the salt form.

### Cocaine Self-Administration

**Apparatus.** Twelve standard operant conditioning chambers described in detail elsewhere (15) were used (Methods in Supplement 1).

**Figure 1:**
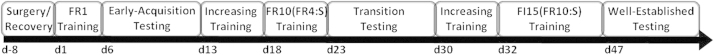
The timeline of self-administration experimentation. Subjects underwent intravenous catheter and central cannulae surgery a week before beginning behavioral training. There were five sessions of fixed-ratio 1 (FR1) training followed by early-acquisition testing. From Days 13 to 17, the response requirement was increased across sessions to the mid-stage training schedule of FR10(FR4:S). Rats remained on that schedule for five sessions before entering mid-stage testing. The response requirement was again increased on Days 30 and 31 to the final second-order training schedule, FI15(FR10:S). Rats were again tested after 15 training sessions from Days 32 to 46 on the final schedule of reinforcement. Late-stage testing began on Day 37. d, day; FI, fixed-interval.

**Procedure.** The timeline of self-administration procedures is shown in Figure 1. Briefly, cocaine self-administration training sessions began 7 days after surgery. Cocaine (.25 mg/infusion; .1 mL/5 sec) was available under a fixed-ratio 1 (FR1) (continuous reinforcement) schedule of reinforcement in which one active lever press resulted in an infusion and initiated a 20-sec timeout. During that 20 sec, the cue-light (conditioned stimulus [CS]) above the active lever was illuminated, the house light was extinguished, and both levers were retracted. Pressing on the inactive lever was recorded to provide an index of general activity but had no programmed consequence. A maximum of 30 cocaine infusions was available at this stage. Active and inactive lever assignment was counterbalanced.

After five training sessions under the FR1 schedule of reinforcement, the dose-dependent effects of striatal dopamine receptor blockade on early-stage cocaine seeking were tested. Bilateral infusions of α-flupenthixol were made into the DLS. These 15-min test sessions [FI15(FR10:S)] instituted a change in contingency in that every active lever press resulted in a 1-sec light CS presentation, and cocaine was only delivered on the first lever press after the 15-min interval (23). Thus, the early performance tests were conducted before and were thus unaffected by self-administered cocaine on these sessions, because they were explicitly assessed for cocaine seeking within the fixed interval rather than a fixed ratio. Each test session was immediately followed by a FR1 cocaine self-administration training session (30 reinforcers over 2 hours), and rats were given a training session between test days so as to confirm and maintain a stable cocaine-taking baseline.

After the tests evaluating the early performance of cocaine seeking, the response requirement was increased across the daily training sessions through the following schedules of reinforcement: FR1; FR3; FR5(FR2:S); FR10(FR2:S); then to FR10(FR4:S). Under each intermediate second-order schedule, completion of the unit schedule (given within parentheses) resulted in a 1-sec CS light presentation; cocaine infusions and the 20-sec timeout were given only upon completion of the overall schedule. Therefore, for the transition-stage assessments, rats had been trained under conditions that promote the association between instrumental responding and conditioned reinforcers: contingent presentations of the cocaine-associated CS occurred after 4 responses (FR4:S); and cocaine was delivered on completion of the 10th set of four lever presses. Rats remained on this schedule for five training sessions before beginning the transition-stage cocaine-seeking tests. During each 15-min test session with α-flupenthixol infusions in the DLS, every four active lever presses continued to result in a 1-sec light CS presentation, and cocaine was only delivered on the fourth lever press after the 15-min interval [i.e., FI15(FR4:S)]. Thus, the transition-stage performance tests were again conducted before and were unaffected by daily self-administered cocaine. Each test session was immediately followed by an FR10(FR4:S) cocaine self-administration training session (30 reinforcers over 2 hours), and rats were given a training session between test days so as to confirm and maintain a stable cocaine-taking baseline.

After completing the tests evaluating cocaine seeking at the transition stage, the response requirements were again increased through daily training sessions across the following schedules of reinforcement: FR10(FR6:S); FR10(FR10:S); and finally to an overall fixed interval (fixed ratio) schedule of FI15(FR10:S) used in previous studies (23,28). During the final FI15(FR10:S) schedule, responding was maintained by contingent presentation of the cocaine-associated CS after 10 responses (FR10:S); cocaine was delivered on completion of the first 10 lever presses after the expiration of each 15-min fixed interval. At this final stage, there was a limit of five available cocaine infusions. Rats were trained under this FI15(FR10:S) schedule of reinforcement for 15 sessions before the well-established, or late-stage, tests were conducted, in which the effects of α-flupenthixol infusions in the DLS were again assessed. The first interval (FI15) of the second-order schedule provides a time period in which no cocaine has been administered, yet rats are actively seeking the drug. Two rats were removed before the final tests, due to faulty catheters. Rats were given at least one session of training under FI15(FR10:S) conditions between each α-flupenthixol infusion test to ensure baseline stable baseline levels of responding.

### Intrastriatal Infusions

For all three testing stages, intrastriatal infusions (.5 μL/side) of α-flupenthixol (0, 5, 10, and 15 μg/infusion in a counterbalanced, Latin-square order of treatment) were made with 28-gauge steel hypodermic injectors (Plastics One, Roanoke, Virginia) lowered to the injection sites 2 mm ventral to the end of the guide cannulae (i.e., DV-5 mm). Bilateral infusions were made over 90 sec with a syringe pump (Harvard Apparatus, Holliston, Massachusetts) and were followed by a 60-sec diffusion period before injectors were removed and obturators were replaced. Test sessions began 5 min later.

### Histology

At the end of the experiment, histology was conducted as described previously (23) (Supplement 1).

### Statistical Analyses

Premature responses in the 5-CSRTT were analyzed with 2-way analyses of variance (ANOVAs) with Session as the within-subject factor, and Group (HI or LI) as the between-subjects factor. Premature responses were then correlated with selected training measures from the 5-CSRTT, and significant correlations were confirmed with between-subject *t* tests.

Recruitment of DLS dopaminergic involvement in cocaine seeking was confirmed with a three-way ANOVA with Stage (early, transition, and well-established), Dose (0, 5, 10, and 15 μg), and Lever (active and inactive) as within-subject factors. The differential recruitment of DLS dopaminergic involvement in cocaine seeking between HI and LI rats was investigated with a three-way ANOVA with planned contrasts (29) with Session (weights on session 2 vs. session 1) and Dose (weights on doses of 10 and 15 µg/side vs. vehicle) as the within-subject factors and Group (HI or LI) as the between-subjects factor. Differences between HI and LI rats for each stage were then investigated with ANOVA with Dose and Lever as within-subject factors. Significant interactions were analyzed further with Tukey’s honestly significant difference (HSD) tests. Significance was set at α = .05.

## Results

### 5-CSRTT

**Figure 2:**
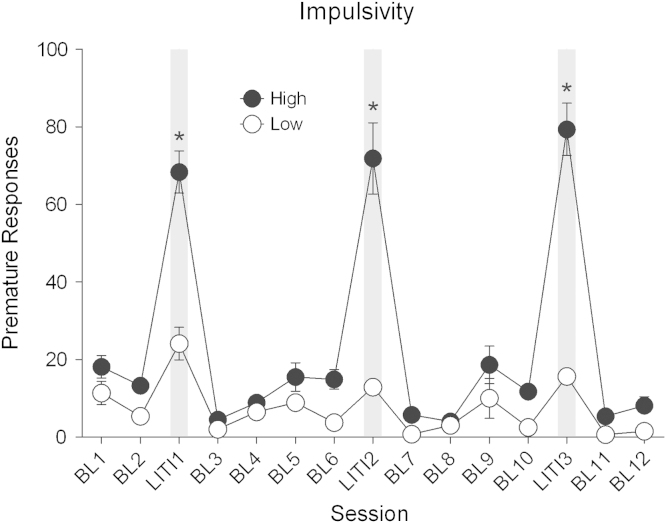
High-impulsive rats are characterized by a high number of premature responses made before the onset of the target stimulus during the long inter-trial intervals (LITIs) but not during baseline (BL) sessions. *Significant difference from low-impulsive rats during the same LITI.

Rats selected as HI (*n* = 8) in the 5-CSRTT displayed greater sensitivity to increased ITI duration than the LI (*n* = 8) rats as supported by increases in premature responses for the three LITI trials for the HI compared with the LI rats (Figure 2) (main effects of Group: F_1,14_ = 65.20, *p* < .001, Session: F_14,196_ = 59.34, *p* < .001, and Group × Session interaction: F_14,196_ = 25.44, *p* < .001). Post hoc analysis revealed that group differences emerged as a result of lengthening the ITI (HSD = 14.477).

**Figure 3:**
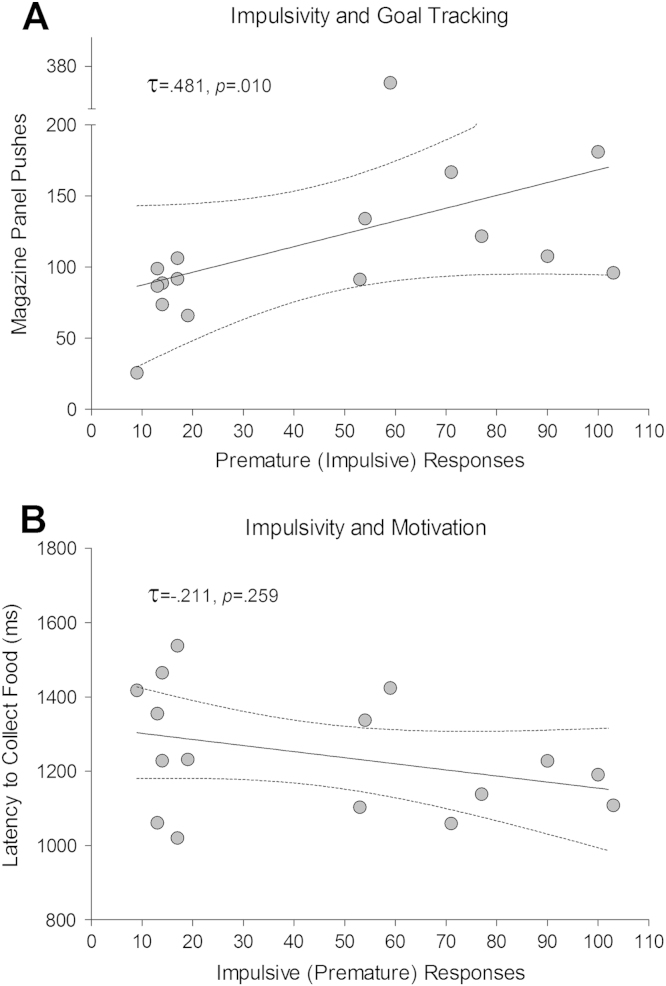
Premature responses during the long inter-trial interval (LITI) sessions were correlated with magazine panel pushes (goal-tracking) **(A)** and latency to collect reinforcers (motivation) **(B)** during training sessions. High-impulsive rats showed higher levels of interaction with the magazine, but not more motivated to obtain the reward, than low-impulsive rats.

Higher impulsivity (measured as the level of premature responses during the last two LITI sessions) was related to greater amounts of goal tracking (measured as panel pushes into the magazine) and latency to collect earned pellets as revealed by a positive relationship between premature responses and panel pushing during training (τ = .481, *p* = .010) (Figure 3A); this was further confirmed by a follow-up *t* test comparing the number of panel pushes in HI and LI rats (*t*_14_ = 2.36, *p* = .033). Impulsivity was not, however, related to motivation for the reinforce, as revealed by both the lack of relationship between the number of premature responses and the latency to collect pellets after a correct trial (τ = −.211, *p* = .259) (Figure 3B) and the absence of a difference in this latter measure between HI and LI rats (*t*_14_ = 1.14, *p* = .273). Baseline behavioral measures recorded during the training sessions immediately preceding LITI 2 and 3 are shown in Table S1 in Supplement 1.

### Histological Assessments

**Figure 4:**
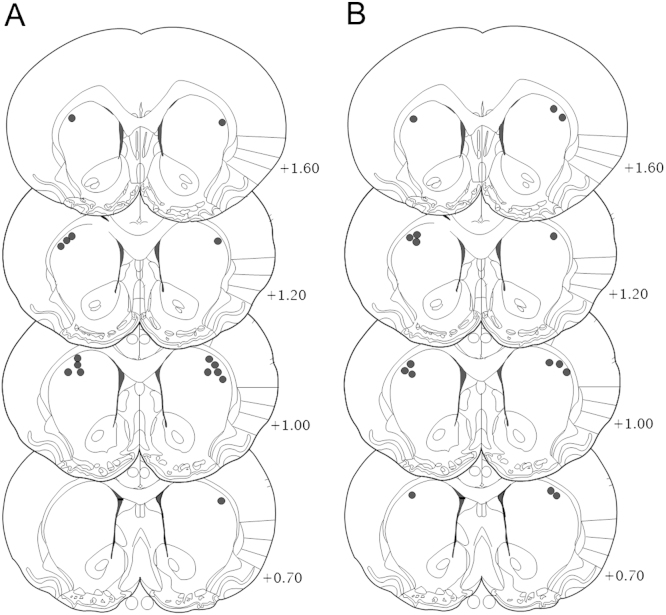
Schematic representations of the localization of injection sites in high-impulsive **(A)** and low-impulsive **(B)** rats with guide cannulae placed in the anterior dorsolateral striatum. Reprinted from Paxinos and Watson (27) with permission from Elsevier, copyright 1998.

All rats had cannulae located bilaterally within the DLS (Figure 4) (27).

### Recruitment of DLS Dopamine Control over Cocaine Seeking

**Figure 5:**
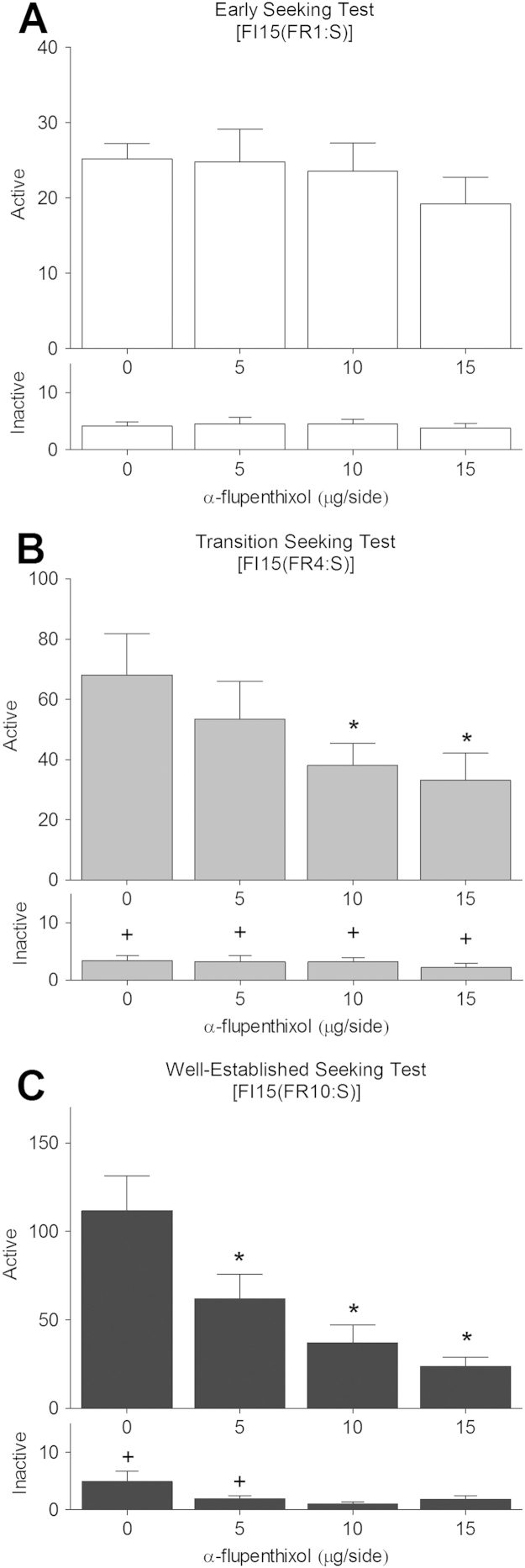
Progressive recruitment of dopamine-dependent dorsolateral striatum control over cue-controlled cocaine seeking. Active and inactive lever presses (±1 SEM) during (cocaine-free) tests of drug seeking with α-flupenthixol injections into the dorsolateral striatum of high- and low-impulsive rats combined at the early **(A)**, transition **(B)**, and well-established **(C)** stages of training. *Significant difference in active lever responding from the 0 μg test. ^+^Significant difference between active and inactive lever responses for each dose tested. FI, fixed-interval; FR, fixed-ratio.

Progressive recruitment of dopamine-dependent DLS processes in the control over well-established, habitual, cue-controlled cocaine-seeking behavior was observed from early to late stage tests as illustrated by the progressive increase in the effect of bilateral intra-DLS α-flupenthixol infusions on active lever presses during the 15-min drug-free cocaine-seeking interval (Stage × Dose × Lever interaction: *F*_6,78_ = 3.50, *p* = .004), confirming our earlier results (15,23). Thus, although dopamine receptor blockade in the DLS was ineffective during the early stage of cocaine seeking (Figure 5A) (effect of Dose: *F*_3,45_ = 1.03, *p* = .389 and Lever × Dose interaction: *F*_3,45_ = 1.06, *p* = .375), it dose-dependently reduced cocaine seeking when performed at the transition stage (Figure 5B) (main effect of Dose, *F*_3,45_ = 3.41, *p* = .025; and a Lever × Dose interaction, *F*_3,45_ = 3.45, *p* = .024). Post hoc analyses revealed that this effect was attributable to the 10- and 15-μg/side doses of α-flupenthixol (HSD = 26.59). When cue-controlled cocaine seeking was well-established, bilateral DLS α-flupenthixol infusions resulted in an even more pronounced decrease in cocaine-seeking responses measured during the 15-min drug-free interval (Figure 5C) (main effect of Dose: *F*_3,39_ = 9.69, *p* < .001 and Lever × Dose interaction: *F*_3,39_ = 9.01, *p* < .001). At this stage, all doses of α-flupenthixol significantly reduced cocaine seeking relative to vehicle (HSD = 40.30).

### Impulsivity Is Associated with a Delayed Transition to DLS Dopamine Control over Cocaine Seeking

**Figure 6:**
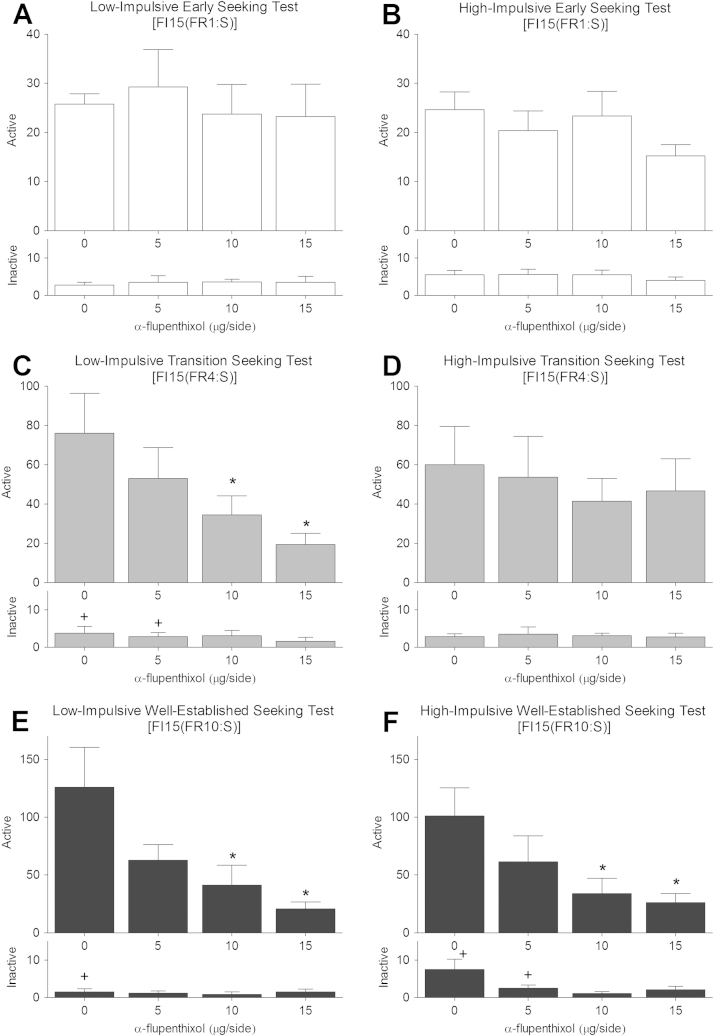
Delayed transition to dorsolateral striatum control over cocaine-seeking behavior in high impulsive rats. Active and inactive lever presses (±1 SEM) during (cocaine-free) tests of drug seeking with α-flupenthixol injections into the dorsolateral striatum of low- and high-impulsive rats at the early (**A**, **B**, respectively), transition (**C**, **D**, respectively), and well-established (**E**, **F**, respectively) stages of training. *Significant difference in active lever responding from the 0 μg test. ^+^Significant difference between active and inactive lever responses for each dose tested. FI, fixed-interval; FR, fixed-ratio.

The progressive recruitment of DLS dopamine control over cocaine seeking observed in the entire population was modulated by impulsivity status. Thus, HI and LI rats displayed different time-courses in their sensitivity to DLS dopamine receptor blockade over the transition from early to well-established, habitual, cue-controlled cocaine seeking (Session × Dose × Group contrasts: *F*_1,12_ = 8.07, *p* < .05). Thus, whereas DLS α-flupenthixol infusions had no significant effect on active lever presses in HI (Figure 6A) and LI rats (Figure 6B) during the early seeking tests (main effects of Dose or Dose × Lever interaction: *F*s ≤ 2.83, *p* ≥ .063), they dose-dependently decreased cocaine seeking in LI rats (Figure 6C) (main effect of Dose: *F*_3,21_ = 3.89, *p* = .023, and a Dose × Lever interaction: *F*_3,21_ = 3.86, *p* = .024) but not in HI rats (Figure 6D) (*F*s < 1) during the transition seeking tests. Post hoc analyses revealed that cocaine seeking in LI rats behavior was decreased after infusions of 10- and 15-μg/side doses of α-flupenthixol relative to vehicle and inactive lever presses (HSD = 40.62).

In the well-established seeking tests, after rats had been trained to seek cocaine under the control of contingent presentations of the drug associated CSs, during the FI15(FR10:S) stage of the second-order schedule, responding was dose-dependently decreased by bilateral infusions of α-flupenthixol into the DLS in both HI and LI rats. LI rats continued to display dose-dependent effects of α-flupenthixol infusions into the DLS (Figure 6E), while this sensitivity to DLS dopamine receptor blockade now emerged in HI rats (Figure 6F) (main effect of Dose: *F*_3,15_ = 5.23, *p* = .011 and *F*_3,21_ = 4.11, *p* = .019, respectively, Dose × Lever interaction: *F*_3,15_ = 5.20, *p* = .012 and *F*_3,21_ = 3.59, *p* = .031, respectively). Thus, the 10 and 15 μg/side doses of α-flupenthixol markedly reduced active-lever presses relative to vehicle such that significant differences between active and inactive lever pressing were no longer observed (HSD = 69.58 and HSD = 55.62 for LI and HI rats, respectively).

Although a shift in the time course of the recruitment of dopamine-dependent DLS control over cue-controlled cocaine seeking was observed between HI and LI rats, the two groups differed neither in the propensity to initiate cocaine self-administration over the five FR1 acquisition sessions (main effect of Session: *F*_4,56_ = 3.124, *p* = .022 but no effect of Group: *F*_1,14_ = 1.606, *p* = .226, or Group × Session interaction: *F* < 1) nor in their performance to the increasing behavioral demands associated with each stage of the establishment of a second-order schedule of reinforcement for the drug. Indeed, no differences were observed in cocaine-seeking responses between HI and LI rats either during the five FR10(FR4:S) sessions that preceded the intermediate stage assessment (all *F*s < 1) or during the FI15(FR10:S) sessions that preceded the late stage assessment (main effect of Group: *F*_1,12_ = 1.367, *p* = .265, and Group × Session interaction: *F*_14,168_ = 1.167, *p* = .305), despite an overall increase in active lever presses over the sessions, indicative of the progressive increase in the influence of contingent presentations on CS on instrumental cocaine-seeking responses over time (main effect of Session: *F*_14,168_ = 1.872, *p* = .033).

## Discussion

Cocaine-induced intrastriatal processes eventually resulting in DLS dopamine-dependent drug-seeking habits (3,14,15,23,30–31) are increasingly considered to be a pivotal mechanism during the development of addiction (16). Although impulsivity characterized by low ventral striatal D2/3 dopamine receptor availability (19) has been identified as a key marker of the individual propensity to switch from controlled to compulsive drug use (18), the ways in which impulsivity and its underlying neural substrates interact with drug-induced intrastriatal adaptations are unknown. According to our earlier speculation (28) and a computational model of addiction based on striatal function (20), the trait of high impulsivity and associated low dopamine D2/3 ventral striatal dopamine receptors (19) has been suggested to facilitate drug-induced recruitment of DLS-dependent habitual control over cocaine-seeking behavior. By contrast, integrative hypotheses suggest that addiction develops when the neurobiological underpinnings of impaired executive, corticostriatal-dependent, inhibitory control, lying at the core of impulsivity, add to and converge with those associated with drug-induced intrastriatal shifts subserving the development of cue-controlled drug**-**seeking habits (6,7,21,32,33).

The findings in the present study support the latter view by providing evidence that increased impulsivity does not facilitate or accelerate the progressive recruitment of dopamine-dependent DLS control over behavior that has been shown to underlie both drug-seeking habits and compulsive cocaine seeking (3,6,15,16,23). Instead, high impulsivity was associated with a delay in striato-striatal neuroadaptations leading to the progressive devolution of control over cocaine seeking to DLS dopamine-dependent processes. This thereby indicates that the interaction between impulsivity and cocaine-induced recruitment of dopamine-dependent dorsolateral striatal control over behavior underlying the eventual transition to compulsive drug seeking (16) might depend upon interactive, co-occurring corticostriatal and striato-striatal processes. It might therefore be speculated that compulsive drug seeking arises from the development of qualitatively aberrant, rigid, maladaptive habits in vulnerable individuals that are characterized by premorbid alterations in corticostriatal-dependent inhibitory control processes.

Thus, in HI rats, there was a shift in the time-course of the effects of bilateral intra-DLS infusions of the dopamine receptor antagonist α-flupenthixol to reduce active lever presses during the 15-min drug-seeking challenge tests. Although DLS dopamine receptor blockade had no effect on cue-controlled cocaine-seeking responses at the early performance test stage, it significantly decreased active lever presses at the later, habitual test stage, the two test stages when there were no significant differences between HI and LI rats. These data, in agreement with our previous work (23), thereby demonstrate that—regardless of differences in impulse control—all subjects eventually develop DLS dopamine-dependent cocaine-seeking habits after protracted drug-seeking performance (3,8,15,23). However, at the intermediate stage of training, cocaine-seeking responses were decreased by DLS dopamine receptor blockade specifically in LI but not HI rats.

This delayed recruitment of the DLS in the control over cocaine seeking suggests that low availability of ventral striatum dopamine D2 receptors might influence drug-induced adaptations underlying the progressive ventral to dorsal striatal shift that occurs in the course of addiction in humans (12,34) and during extended periods of cocaine self-administration in nonhuman primates (8,9,11,35) and rats (10). We and others have suggested that this ventral to dorsal striatal shift depends upon the dopamine-dependent ascending spiraling circuitry (36,37) functionally linking the ventral with the dorsolateral striatum (13,15,31,38), even though the mechanisms whereby this circuitry is recruited remain to be established. Added to the recent demonstration that the progressive cocaine-induced ventral to the dorsal striatum decrease in dopamine D2 receptors and messenger RNA (mRNA) levels demonstrated in primates (39–41) and rats (10) is also delayed in HI as compared with LI rats (10), despite lower baseline levels of D2 mRNA in the nucleus accumbens shell and dopaminergic neurons of the former (10), the present results suggest that low D2 receptor availability in the ventral striatum retards intra-striatal cocaine-induced plasticity processes. This is consistent with the demonstration that individual vulnerability to develop addiction-like behavior for cocaine, that we have demonstrated to be highly predicted by high impulsivity (18), is associated with impaired cocaine-induced plasticity in the ventral striatum (42).

Although protracted cocaine exposure results in marked decreases in striatal D2 dopamine receptor and mRNA levels, an adaptation suggested to contribute to the development of addiction (39,43–45), cocaine self-administration in HI rats that display spontaneous low D2 mRNA and receptor levels in the ventral striatum results in a normalization of D2 receptor levels (46) that parallels a reduction in impulsivity. This observation therefore suggests that the potential delay in dorsal striatal recruitment after cocaine exposure observed in HI rats might be attributed to cocaine-induced remediation of low D2 dopamine receptors in the ventral striatum and the associated impulsivity that occurs early on after cocaine self-administration. Indeed, this hypothesis is supported by a recent micro positron emission topography study in LI and HI rats (46). This has important implications at the psychological level in that it suggests that, for HI rats, instrumental actions for cocaine might remain goal-directed for longer than in LI rats, a consequence partly determined by a dopamine deficiency state in the ventral striatum. This is consistent with the observation that HI rats are more focused on a food goal than LI rats, spending more time at the food delivery magazine when trained in the 5-CSRTT. Moreover goal-trackers in a Pavlovian conditioned approach task motivated by food were more impulsive in a delay discounting task than sign-trackers (47), a dimension of impulsivity that is also expressed by HI rats selected in the 5-CSRTT (48). These observations indicate that impulsivity is associated with a dominance of goal-directed behavior during early experience in instrumental and Pavlovian tasks.

The present results show that the psychological mechanisms whereby impulsivity and habits contribute to addiction do not depend upon a facilitation of the development of the latter by the former. However, it is pivotal to dissociate the propensity to develop habits, which in itself is not an aberrant process, from the inability to regain control over maladaptive habits that have become inflexible, such as those that are seen in addicted individuals who compulsively seek and take drugs. This further suggests that vulnerability to addiction does not lie in the propensity of an individual to develop habits but instead in the rigid nature of drug-seeking habits and the inability of an individual to regain control over these maladaptive habits. This inflexibility of drug-seeking habits might stem from either cortical (49) or striatal components of weak inhibitory control or in the persistence of aberrant neurobiological adaptations that have accumulated during the recruitment of dorsolateral striatal control over behavior to overcome the apparent lack of striatal neuroplasticity that characterizes HI rats (10).
