# Supplementary material for: Increased Impulsivity Retards the Transition to Dorsolateral Striatal Dopamine Control of Cocaine Seeking
Source: Biol Psychiatry. 2014 Jul 1;76(1):15–22. doi: 10.1016/j.biopsych.2013.09.011 (PMC4064115; doi:10.1016/j.biopsych.2013.09.011)
Supplement: Supplementary file 1 — Supplementary Material [file mmc1.pdf]

Increased Impulsivity Retards the Transition to Dorsolateral Striatal Dopamine Control of Cocaine Seeking

Supplemental Information

Supplemental Methods and Materials

Subjects

Forty male Lister Hooded rats (Charles River Laboratories, Kent, UK) weighing approximately 300 g on arrival were habituated for one week in a 12-hr reverse light:dark cycle (lights off at 0700). Throughout the experiments, rats were fed 20 g chow per day given within 2 hrs of completing each daily training session. Water was freely available in the home cages. Rats were housed four to a cage during behavioral training on the 5-choice serial reaction-time task, then individually during cocaine self-administration training. Experiments were conducted during the dark phase, 5-7 days per week, and in accordance with the United Kingdom 1986 Animals (Scientific Procedures) Act.

5-Choice Serial Reaction Time Task Apparatus

We used twelve chambers, each placed within individual ventilated sound- and light-attenuating boxes. The rear wall of the chamber was curved with five contiguous 2.5 x 2.5cm apertures equipped with photocell beams to detect nose-poke responses. A 3W stimulus light was located at the rear of each of the five apertures. On the opposite side of the chamber, a magazine connected to a pellet dispenser allowed the automatic delivery of 45-mg food pellets (Noyes dustless pellets, Research Diets, UK). Subjects gained access to the food magazine by pushing a panel monitored by a micro-switch. Operant chambers were controlled using the Whisker server and control system (Cardinal & Aitken, http://www.whiskercontrol.com).

Surgeries

Rats were anaesthetized with an intraperitoneal injection of ketamine hydrochloride (100 mg/kg; Ketaset; Fort Dodge Animal Health Ltd, Southampton, UK) and xylazine (12 mg/kg; Rompun; Bayer, Wuppertal, Germany). They were then implanted with an intravenous catheter (CamCaths, Ely, UK), made from silastic tubing attached to a 22-gauge steel cannula at its external end, into the right jugular vein. The tubing ran subcutaneously over the shoulder and exited between the scapulae where it was sutured in place. Following implantation, rats were positioned in a stereotaxic frame (David Kopf Instruments, Tujunga, CA) and were implanted bilaterally with 22-gauge guide cannulae (Plastics One, Roanoke, VA) positioned to lie 2 mm above the dorsolateral striatum (AP +1.2, ML ±3, DV -3; (1); AP

and ML coordinates measured from bregma, DV coordinates from the skull surface, incisor bar at -3.3 mm; (2)). Stainless steel screws tapped into the skull were anchors for the dental acrylic that held the cannulae in place. Obdurators (Plastics One) were kept in the cannulae to maintain patency. From the day before to seven days after surgery, rats were treated subcutaneously with 10 mg/kg of the antibiotic Baytril (Bayer). Catheters were flushed daily throughout the experiment with 0.2-0.4 ml of sterile saline mixed with heparin (20 U/ml; Wockhardt UK Ltd, Wrexham, UK) to maintain patency.

Self-Administration Apparatus

Each of twelve operant conditioning chambers (29.5 x 32.5 x 23.5 cm; Med Associates, St. Albans, VT) was housed in a sound- and light-attenuating cubicle fitted with a ventilation fan. Chambers were equipped with 4-cm wide retractable levers 8 cm above the grid floor and 12 cm apart. Above each lever was a white cue light (2.5 W, 24 V), and a white houselight (2.5 W, 24 V) was at the top of the opposite wall. Chamber sidewalls were aluminum; the ceiling, front, and back walls were clear polycarbonate. Each chamber contained a spring leash attached to a swivel connected to a balanced metal arm secured outside of the chamber. Tygon tubing extended from the catheter through the leash to the swivel and from the swivel to a 10-ml syringe mounted on a syringe pump (Semat Technical, Herts, UK) located outside each cubical. Personal computers with Whisker software (Cardinal and Aitken, http://www.whiskercontrol.com) controlled infusions and light presentations and recorded lever presses.

Histology

Rats were euthanized with an overdose of sodium pentobarbital (300 mg; Dolethal; Vétoquinol UK Ltd, Buckingham, UK) then perfused transcardially with isotonic saline followed by 10% neutral buffered formalin. Brains were extracted and transferred to a 20% sucrose solution in 0.01 M PBS for approximately 24 hrs before sectioning at 60 µm. Every third slice was mounted and stained with Cresyl Violet. Cannulae placements in the dorsolateral striatum were verified using a light microscope.

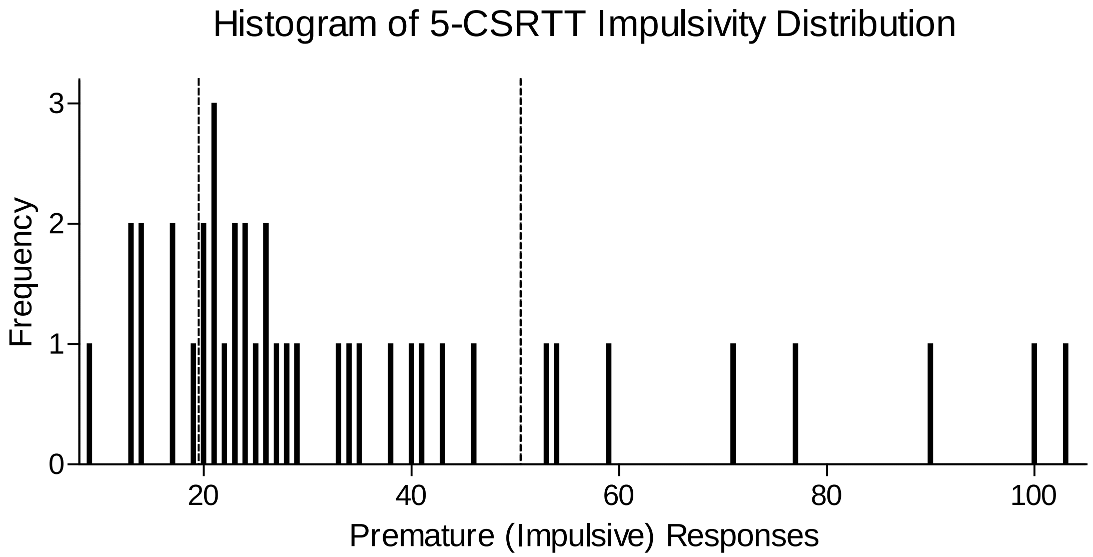

Figure S1. The distribution of premature responses in the 5-Choice Serial Reaction Time Task (5-CSRTT) is shown. Dashed lines indicate the <20 premature responses for the Low-Impulsivity criterion and the >50 premature responses for the High-Impulsivity criterion.

Table S1. Additional measures from the 5-CSRTT assessment. The average of the scores from the training trial immediately preceding LITI2 and LITI3 are shown (±SEM) for high and low impulsive phenotypes selected based on the average of premature responses on LITI2 and LITI3. Between-group *t*-tests were conducted for each measure.

	**High Impulsive**	**Low Impulsive**	**Statistics**
Premature Responses	13.31 ± 1.41	3.13 ± 0.81	$t(14) = 6.27$, $p < .001$
Magazine Panel Pushes	158.34 ± 32.06	79.73 ± 8.96	$t(14) = 2.36$, $p = .033$
Collection Latency (ms)	1198.62 ± 44.59	1289.66 ± 66.16	$t(14) = 1.14$, $p = .273$
Correct Trials	67.25 ± 2.17	68.94 ± 3.06	$t(14) = 0.45$, $p = .660$
Incorrect Trials	26.25 ± 2.46	20.13 ± 1.73	$t(14) = 2.04$, $p = .061$
Omissions	6.50 ± 2.67	10.94 ± 5.06	$t(14) = 2.19$, $p = .046$

5-CSRTT, 5-Choice Serial Reaction Time Task; LITI, long inter-trial interval.

Supplemental References

1. Belin D, Everitt BJ (2008): Cocaine-seeking habits depend upon dopamine-dependent serial connectivity linking the ventral with the dorsal striatum. *Neuron* 57: 432–441.

2. Paxinos, G, Watson C (1998): *The rat brain in stereotaxic coordinates.* San Diego, CA: Academic Press.
